# Quantitative Analysis of 3-Monochloropropane-1,2-diol in Fried Oil Using Convolutional Neural Networks Optimizing with a Stepwise Hybrid Preprocessing Strategy Based on Fourier Transform Infrared Spectroscopy

**DOI:** 10.3390/foods14101670

**Published:** 2025-05-09

**Authors:** Xi Wang, Siyi Wang, Shibing Zhang, Jiping Yin, Qi Zhao

**Affiliations:** 1 State Key Laboratory of Marine Food Processing and Safety Control, School of Food Science and Technology, Dalian Polytechnic University, Dalian 116034, China; wangxi9215@icloud.com (X.W.); www011478@163.com (S.W.); 13470306550@163.com (S.Z.); 2 Information Technology Center, Dalian Polytechnic University, Dalian 116034, China; chinayinjp@dlpu.edu.cn

**Keywords:** 3-monochloropropane-1,2-diol, Fourier transform infrared spectroscopy, data preprocessing, convolutional neural network, rapid detection

## Abstract

As one kind of ‘probable human carcinogen’ (Group 2B) compound classified by the International Agency for Research on Cancer, 3-MCPD is mainly formed during the thermal processing of food. Tedious pretreatment techniques are needed for the existing analytical methods to quantify 3-MCPD. Hence, a nondestructive sensing technique that offers low noise interference and high quantitative precision must be developed to address this problem. Following this, Fourier transform infrared spectroscopy association with an convolutional neural network (CNN) model was employed in this investigation for the nondestructive quantitative measurement of 3-MCPD in oil samples. Before building the CNN model, NL-SGS-D_2_ was utilized to enhance the feature extraction capability of model by eliminating the background noise. Under the optimal hyperparameter settings, calibration model achieved a determination coefficient (R^2^_C_) of 0.9982 and root mean square error (RMSEC) of 0.0181 during validation, along with a 16% performance enhancement enabled by the stepwise hybrid preprocessing strategy. The LODs (0.36 μg/g) and LOQs (1.10 μg/g) of the proposed method met the requirements for 3-MCPD detection in oil samples by the Commission Regulation issued of EU. The method proposed by CNN model with hybrid preprocessing was superior to the traditional model, and contributed to the quality monitoring of edible oil processing industry.

## 1. Introduction

3-Chloro-1,2-propanediol (3-MCPD) has been recognized as a ‘probable human carcinogen’ (Group 2B) by the International Agency for Research on Cancer, and the generation of this contaminant predominantly associated with processing and handling techniques [1]. As known from animal experiments, the free monomer of 3-MCPD was found to possess carcinogenic characteristics, such as certain organic dysfunctions [2]. A maximum permissible of 3-MCPD in oil was 750 µg/kg mandated by the Commission Regulation issued in the European Union at 2021 [3]. Consequently, the establishment of a detection method for 3-MCPD was deemed highly necessary.

The conventional approach for the quantification of 3-MCPD content in fats and oils relied on Gas Chromatography–Mass Spectrometry (GC-MS) by the official method described in AOCS [4], which involved complex pre-treatment and derivatization procedures. Therefore, the development of a faster and more cost-effective detection method was recognized. Recently, emerging techniques such as electrochemical analysis [5], biosensors [6], fluorescence colorimetry [7] and quantitative infrared spectroscopy [8] have been utilized. Among them, Fourier Transform Infrared (FTIR) spectroscopy had demonstrated great research potential due to its simplicity of operator, non-destructive nature, and reagent-free environmental friendliness. Based on the fingerprint spectral information in FTIR spectroscopy, the 3-MCPD can be quantified by the constructed mathematical models to correlate the peak intensity with concentration labels [8]. However, challenges were also encountered in the practical application of FTIR quantification analysis, such as instrument noise, low sensitivity, and matrix effects, which reduced the performance of model.

To address the issues of background noise and signal interference in spectroscopic analysis, mathematical modeling methods such as partial least squares regression (PLSR), support vector regression (SVR), random forest (RF), and convolutional neural networks (CNN) had been developed [9]. However, PLSR and SVR exhibited insufficient capacity in handling nonlinear problems [10], while RF struggled to capture variations between adjacent features [11]. In contrast, CNN models were recognized as representative mathematical frameworks due to their multi-scale feature learning through hierarchical convolutions [12]. As a deep learning tool composed of input layers, convolutional layers, pooling layers, and fully connected layers, CNNs are well-suited for analyzing structured data. CNN models have been successfully applied to the quantitative detection of behenic acid [13] and aflatoxin B1 [14] in edible oils. The FTIR spectral feature correlations of 3-MCPD align with the feature extraction mechanisms of CNN models. Meanwhile, the complex nonlinear features of 3-MCPD spectral data were suitable multi-layer processed by hierarchical nonlinear fitting architecture in CNN. Nevertheless, the analytical accuracy was significantly compromised by spectral overlap, which prevented reliable quantification of trace analytes and ultimately led to poor CNN model generalizability [15]. Therefore, mitigating spectral overlap was imperative to enhance the quantification reliability in CNN models for complex matrices.

Data preprocessing was served as an effective strategy to mitigate spectral overlap and achieve noise reduction and feature signal enhancement [16]. Distinct performance characteristics was exhibited in different preprocessing methods. Up to now, preprocessing methods including max–min normalization (NL), Savitzky–Golay smoothing (SGS), standard normal variate (SNV), multiplicative scattering correction (MSC), and derivative processing has been implemented to eliminate the external noise [17]. Nevertheless, due to the compositional complexity of the 3-MCPD sample, the effect of noise reduction was limited by the application of individual approaches [18]. Based on the above, a hybrid data preprocessing strategy was developed to address signal overlap and matrix interference in complex sample compositions [19]. Hence, a stepwise hybrid data preprocessing strategy combined with CNN has been proposed to enhance modeling performance [20] and applied in the analysis of Cd in peanut oil [21], lard in butter [22], and chlorpyrifos residues in corn oil [23]. However, limited studies were conducted on 3-MCPD in edible oils.

In summary, a CNN quantitative model based on data with a stepwise hybrid preprocessing strategy was constructed to analysis 3-MCPD in edible oils. Firstly, different data processing methods were compared to form a stepwise preprocessing strategy to eliminate irrelevant noise. Subsequently, the optimal CNN architecture configuration parameter was optimized to extracted multiscale discriminative fingerprint features. Finally, the trained model was applied to frying oil, and the accuracy was assessed by the coefficient of determination (R^2^) and root mean square error (RMSE).

## 2. Materials and Methods

### 2.1. Reagents and Sample Preparation

Ethyl acetate, hexane, anhydrous ethanol, sodium chloride, anhydrous sodium sulfate, diatomaceous earth, 3-chloro-1,2-propanediol (3-MCPD, purity > 95%) and 3-chloro-1,2-propanediol-d_5_ (d_5_-3-MCPD, purity > 95%) were purchased from McLean Company (Shanghai, China). Heptafluorobutyryl imidazole (HFBA, purity > 97%) was obtained from Aladdin Company (Shanghai, China). Sunflower oil and fresh chicken was procured from local markets.

The standard working solutions were prepared according to the AOCS standard method for standard curve construction [24]. Meanwhile, samples with 3-MCPD concentrations ranging from 0.1 μg g^−1^ to 1.5 μg g^−1^ were prepared using sunflower oil as the solvent for model training and testing.

For model validation samples, fresh chicken had been cut into 100 g pieces. The frying process was conducted using a commercial fryer (Aituo, Model 81A, Guangzhou, China). First, 3.0 L of sunflower oil had been heated to 160 °C within 10 min. Then, the temperature was gradually increased by 20 °C each hour to 200 °C. An initial oil sample was collected prior to the start of frying, and subsequent samples (15 mL each) were obtained at 20 min intervals throughout the frying process. Each sampling was performed in triplicate in parallel. Frying continued for 4 days without the addition of more oil. At the end of frying, the fryer was turned off and allowed to cool to room temperature. All samples were stored at −80 °C and derived with HFBA for further testing.

### 2.2. Spectral Data Collection

Full FTIR spectra of the samples were obtained using a Fourier transform infrared spectrometer (Shimadzu, IRTracer-100, Kyoto, Japan) combined with an infrared liquid cuvette. The instrument was operated in transmission mode, and the sample interferogram was collected within a wavenumber range of 4000–400 cm^−1^. A total of 64 scans were performed for each acquisition, with a resolution of 4 cm^−1^. The air background was scanned before the samples were scanned. Hexane was used to clean the liquid pool after each sample scan. Three measurements were taken in parallel for each sample measurement, and the average value was recorded [25].

### 2.3. Data Processing

#### 2.3.1. Full Factorial Design

Background noise and stray light interference were included in the spectrum along with sample-related information. Full factorial design was proposed to find the optimal preprocessing strategy for noise reduction [26]. To eliminate these interferences, six spectral preprocessing techniques and their combinations were explored using full factorial analysis.

#### 2.3.2. Max–Min Normalization

Max–min normalization (NL) was one of the mathematical tools used in spectroscopy to eliminate or reduce errors caused by measurement conditions or sample properties. NL was a method within normalization techniques that mapped the entire dataset to a specified range. The scale of variations among data was adjusted to the same level by this method, thereby accelerating the model training process in deep learning. This approach also prevented weight bias caused by scale differences. The formula for this method is as follows:

(1) $$X^{\prime }=\frac{X-{\mathrm{Min}}_{X}}{{\mathrm{Max}}_{X}-{\mathrm{Min}}_{X}}$$

where X is the original data, Min_X_ and Max_X_ are the minimum and maximum values of the original data, and X′ is the normalized data [27]. In this study, the y-axes of all spectra were normalized to the range [10, 100].

#### 2.3.3. Savitzky–Golay Smoothing

Savitzky–Golay smoothing (SGS) is a mathematical tool widely employed for data smoothing and differential derivation in spectral analysis and chemical applications. The algorithm applied a local polynomial fit around each data point to approximate a smoother signal. This method was used to smooth the data while preserving the shape and characteristics of the signal more effectively. It was calculated as follows:

(2) $$Y_{j}^{*}=\frac{\sum_{i-=-m}^{i=m}C_{i}Y_{j+i}}{N}$$

where Y is the original spectral data, $Y_{j}^{*}$ is the filtered spectral data, C_i_ is the convolution coefficient of the ith spectral value of the filter within the filter window, i is the offset within the window, N is the number of convolution integers, and m is the half-width of the window [28]. In this study, the SGS algorithm was implemented using a first-order polynomial fitting, with the window width set to 5.

#### 2.3.4. Derivative

The derivative was a key algorithm for enhancing spectral data details, eliminating background noise, and improving resolution. By performing derivative operations on the original spectrum, subtle changes were highlighted, making the data more suitable for the analysis of complex sample matrices. It also improved the detection sensitivity of target components. The fundamental formulas for the first derivative (D_1_) and second derivative (D_2_) were as follows:

(3) $$D^{n}A=\frac{d^{n}A}{d\lambda^{n}}$$

where A represents transmittance, λ denotes the corresponding wavenumber, and n is the order of the derivative [29].

#### 2.3.5. Other Methods and Data Analysis

In addition to the methods mentioned above, MSC and SNV were also employed in this study. MSC was mainly used to remove the effects of sample scattering on the data. It standardizes the spectral baseline and eliminated the linear component to obtain the final result [30]. SNV was used to remove the scattering effects caused by the different shapes and sizes of samples. Its core idea was to eliminate the interference factors in the spectrum by introducing a standardization process [31].

The spectral data were all processed using licensed The Unscrmbler X 10.6.4 (Camo Software, Oslo, Norway), and the operation of normalizing the spectral information in vertical coordinates was performed using licensed OMNIC 7.3 (Thermo Fisher Scientific, Waltham, MA, USA).

### 2.4. Model Building

#### 2.4.1. Data Splitting

All data were randomly segmented before being used as inputs to the model, and the use of 5-fold cross-validation was used to improve the reliability of the results.

#### 2.4.2. Partial Least Squares Regression

PLSR is a linear modeling approach commonly used in spectral detection that identifies relationships between variables by reducing a large number of independent variables into a new set of latent variables. This mathematical relationship could then be used to predict and analyze the samples under investigation. The core equation is as follows:

(4) $$Y=TQ^{T}+F$$

where Y is the dependent variable matrix, T represents the projection of samples in the latent variable space, Q denotes the loading matrix describing how latent variables explain the variability in Y, and F is the residual matrix of Y unexplained by the model [32]. In this study, the maximum number of principal components for the PLSR model was set to 20.

#### 2.4.3. Random Forest

RF was an integrated learning method that improves the performance of classification or regression tasks by combining multiple decision trees. Eventually, all the constructed decision trees were pooled together to form a random forest model. The core equation is as follows:

(5) $$Y=\frac{1}{B}\sum_{b=1}^{B}T_{b}(X;\theta_{b})$$

where B is the total number of decision trees, T_b_(X;θ_b_) denotes the predicted output of the bth decision tree, X represents the input features, θ_b_ corresponds to the randomized parameters for the bth tree, and Y is the final ensemble prediction [33]. In this study, the number of decision trees was optimized to 100.

#### 2.4.4. Support Vector Regression

SVR was a machine learning technique derived from statistical learning theory and was based on structural risk minimization. SVR core lay in using the principle of the maximum margin to determine the optimal hyperplane for accurate classification of data. The radial basis function (RBF) used in this study is defined as follows:

(6) $$K(X_{i},X_{j})=\exp(-\gamma {\left\|X_{i}-X_{j}\right\|}^{2})$$

where K(X) is the kernel function, X_i_, X_j_ are feature vectors, γ is Gaussian function, exp(x) is the exponential function that maps distances to a bounded range [34]. In this study, the Gaussian function was employed as the kernel function, and the model structure was optimized using the ISDA algorithm.

#### 2.4.5. Convolutional Neural Networks

Convolutional neural networks (CNNs) are a classical deep learning structure based on convolutional theory, and consist of input, convolution, pooling, and other related structures. Hyperparameters such as the number of convolutional layers, optimizer function, batch size, weight decay, and number of iterative layers were selected during the training process. The convolutional operation process was as follows:

(7) $$x_{i}^{l}=f\left(\sum_{j=1}^{m^{l-1}}x_{i}^{l-1}w_{i,j}^{l}+b_{i}^{l}\right)(i=1,\;\ldots ,\;m^{l})$$

where x^l^_i_ is the ith out feature on the lth layer, x^l-1^_i_ is the jth output feature on the (l − 1)th layer, w^l^_i,j_ is the weight vector of the convolution kernel between the ith feature on the lth layer and jth feature on the (l − 1)th layer, b^i^ is the bias, m^l^ is the number of features on the lth layer, and f(x) s the activation function of convolutional neurons [35].

### 2.5. Performance Evaluation

The model performance evaluation was primarily based on the coefficient of determination (R^2^), the root mean square error of calibration (RMSEC), the root mean square error of validation (RMSEV), and the root mean square error of prediction (RMSEP), all of which provided a reference for the agreement between predicted and actual values. Higher accuracy was indicated by a higher coefficient of determination (closer to 1) and a lower RMSEV (closer to 0). The determination coefficient and RMSE metrics were derived through application of the equations below:

(8) $$R^{2}=\frac{\sum_{1}^{N}{\left(Y_{p}-\bar{Y_{T}}\right)}^{2}}{\sum_{1}^{N}{\left(Y_{T}-\bar{Y_{T}}\right)}^{2}}$$

(9) $$\mathrm{RMSE}=\sqrt{\frac{1}{N}\sum_{1}^{N}{\left(Y_{T}Y_{p}\right)}^{2}}$$

where N is the number of the entire dataset, Y_T_ denotes the true value of the sample substance, and Y_P_ denotes the predicted value of the sample substance, and denotes the average of all true values.

### 2.6. 3-MCPD Quantification by GC-MS

The operational procedures were all derived from the official AOCS method Cd29c-13 by used GC-MS [24]. For the samples to be tested, a 4 g sample of the formula, 0.02 mL d5-3-MCPD internal standard (10 mg L^−1^) was added, and 4 g of 0.2 g mL^−1^ NaCl solution was added and sonicated and mixed for 5 min. Subsequently, anhydrous sodium sulfate was added to the solution to absorb water. The mixture was allowed to stand for 10 min and then filtered, and the filtrate was evaporated to 0.5 mL at 35 °C. The solution was then dried with 2 mL of n-hexane. Afterwards, the residue was dissolved with 2 mL of hexane and 0.04 mL of heptafluorobutyryl imidazole was added to the system using a gas-tight needle, sealed, and derivatized at 70 °C for 20 min, and then immediately removed and cooled to room temperature. The reaction was stopped by adding 2 mL of 0.2 g mL^−1^ NaCl solution, and the upper organic phase was taken and transferred to a gas chromatography vial after standing and stratifying, ready for detection.

The samples (0.001 mL) were detected using an Agilent GC-MS instrument (Agilent Technologies, 7890-5975C, Santa Clara, CA, USA), equipped with a mass-selective detector. A HP-5MS capillary column (30 m × 0.25 mm × 0.25 μm, Agilent Technologies, Santa Clara, CA, USA) was utilized. The injection port temperature was set at 250 °C. The solvent delay time in the analysis was 5 min, and the programmed temperature increase was 50 °C held for 1 min, increased to 90 °C at 2 °C min^−1^, and then increased to 270 °C at 40 °C min^−1^ and held for 5 min. The helium was flowed at a rate of 1 mL min^−1^.

## 3. Results and Discussion

### 3.1. Spectral Analysis

**Figure 1:**
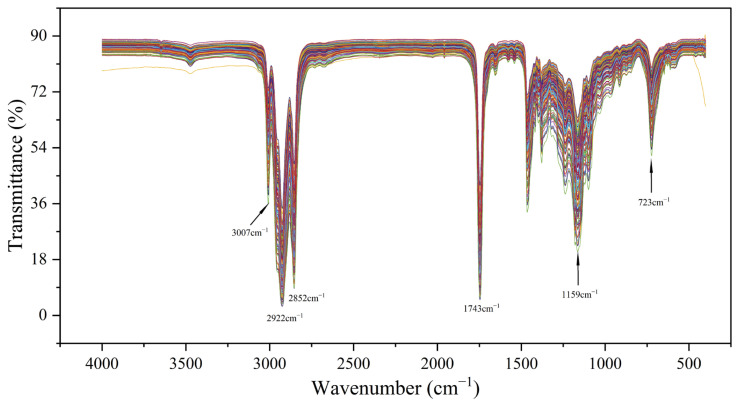
The raw FTIR spectra at 400 cm^−1^ to 4000 cm^−1^ of edible oil with 3-MCPD at 0.1–1.5µg g^−1^.

The FTIR spectra of all spiked samples were systematically recorded as shown in Figure 1, with consistent absorption features observed across all oil samples [36]. Different colors lines were utilized to denote distinct sample spectral curves. The characteristic peak at 1159 cm^−1^ was attributed to C-O ester bond stretching vibrations. Similarly, the absorption band at 1743 cm^−1^ was assigned to C=O double bond stretching vibrations. The spectral signatures at 2852 cm^−1^ and 2922 cm^−1^ were identified as stretching vibrations of methylene (-CH_2_-) and methyl (-CH_3_) groups, respectively. Furthermore, prominent C-H bond stretching vibrations were detected within the 2800–3000 cm^−1^ [37] region. Notably, a distinct absorption band corresponding to C-Cl bond stretching vibrations was observed in the 700–800 cm^−1^ range. This region was recognized as one of the characteristic fingerprints of 3-MCPD, and could serve as a supplementary indicator for quantification [25].

### 3.2. Data Preprocessing and Model Development

#### 3.2.1. Impact of Data Preprocessing on Spectral Profile

**Figure 2:**
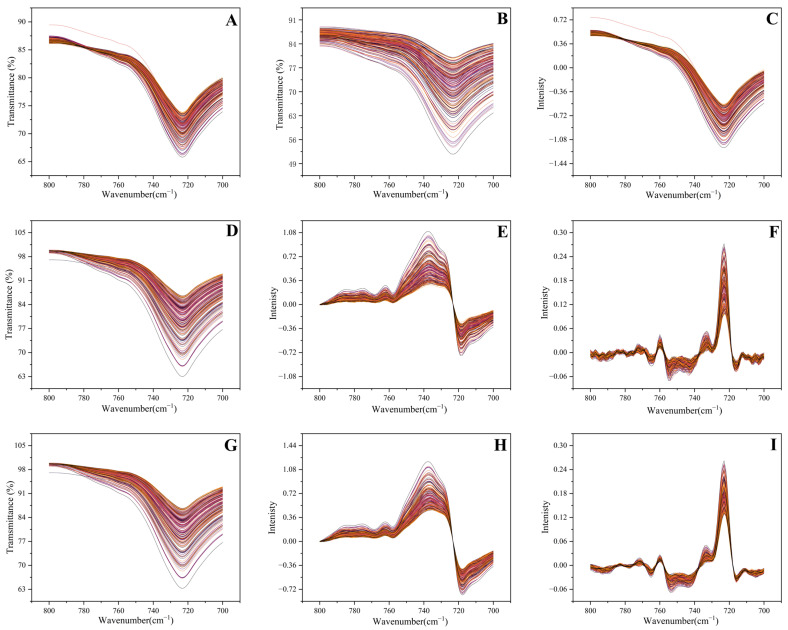
FTIR spectra of 3-MCPD exhibiting characteristic absorption bands (700–800 cm^−1^) across different preprocessing methods. (**A**) MSC, (**B**) SGS, (**C**) SNV, (**D**) NL, (**E**) D_1_, (**F**) D_2_, (**G**) NL-SGS, (**H**) NL-SGS-D_1_, (**I**) NL-SGS-D_2_.

The spectral shape was significantly altered by the preprocessing methods detailed in Table 1. For convenient display, a local spectral region (700–800 cm^−1^) corresponding to the characteristic absorption of 3-MCPD was selected to illustrate the impact of preprocessing on spectral features, with the results presented in Figure 2. Although the spectral noise was smoothed by SGS and NL processing to some extent, the degree exhibited for the characteristic spectral features was insufficient negligible alterations (Figure 2B,D). Despite the scattering effect being eliminated by the application of MSC and SNV, disadvantages like matching shape trends still existed in the processed spectra (Figure 2A,C). While the spectral features were significantly enhanced by derivative processing, residual noise was still appeared in spectral clutter (Figure 2E,H). These results demonstrated that single preprocessing methods had insufficient noise elimination capabilities, aligning with prior studies [19].

The morphological changes of the full spectrum under different preprocessing steps, providing a preliminary observation of characteristic variations, are displayed in Figures S1–S3. Among all preprocessing methods, the hybrid approach integrating NL, SGS and D_2_ (NL-SGS-D_2_) demonstrated the most significant improvement in spectral quality compared to other approaches. The progressive application and the cumulative spectral transformations of the preprocessing method were systematically illustrated in Figure 2G–I. Through the stepwise stacking of the method, spectral baseline drift and noise interference were progressively eliminated, and spectral features were gradually accentuated. The above findings demonstrated that the hybrid preprocessing strategy enhanced spectral features, thereby improving the 3-MCPD quantitative regression model.

#### 3.2.2. Performance Comparison of Different Preprocessing Methods

**Figure 3:**
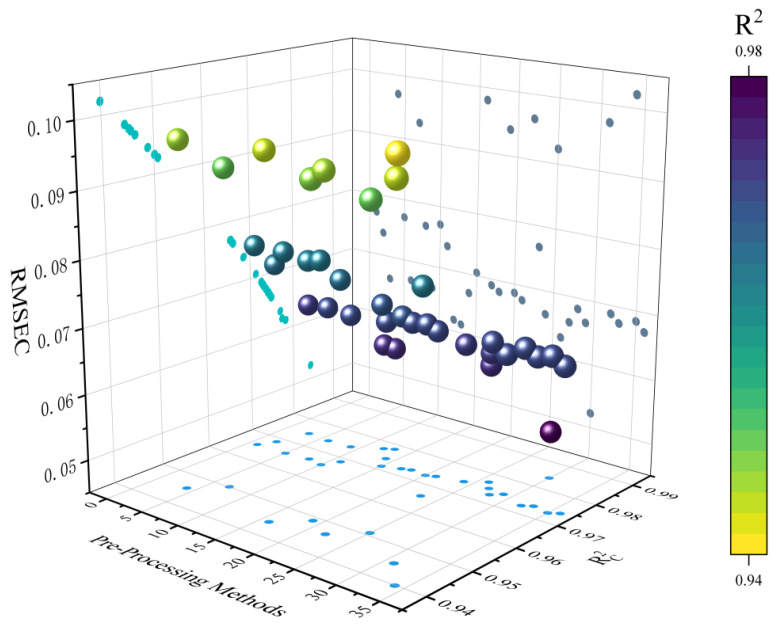
Three-dimensional representation of model performance across hybrid preprocessing method. The X-axis denotes method number, the Y-axis represents the values of R^2^c, and the Z-axis corresponds to the values of RMSEC.

The modeling efficacy of different stepwise hybrid preprocessing methods including R^2^_C_ and RMSEC in PLSR are detailed in Figure 3, and performance specifics are provided in Table S1. The model without preprocessing achieved an R^2^_C_ of 0.9656 and an RMSEC of 0.0771. All the preprocessing methods, except SGS-SNV, NL-MSC, MSC, NL-SGS-MSC, SGS-MSC, and NL-SGS-SNV, were found to increase model performance. Specifically, the model after MSC processing exhibited R^2^_C_ and RMSEC values of 0.9456 and 0.0970. This was attributed to the fact that although the MSC method enhanced spectral quality, the shape trends of the processed spectra were altered. Consequently, additional noise was learned by the model, which negatively impacted its performance. As for D_1_ preprocessing, the model’s R^2^_C_ was increased to 0.9685, and the RMSEC was reduced to 0.0738. D_2_ preprocessing further improved the model performance, with R^2^_C_ and RMSEC values of 0.9746 and 0.0663. These results demonstrated that critical spectral information was moderately enhanced through derivative preprocessing.

Among all preprocessing methods, the NL-SGS-D_2_ hybrid preprocessing strategy achieved the best performance with an R^2^_C_ of 0.9842 and an RMSEC of 0.0523. This phenomenon could be attributed to the NL-SGS-D_2_ preprocessing method effectively eliminating interference from sample thickness and high oil viscosity-related factors (e.g., inhomogeneous light scattering). This finding was consistent with previous studies [25]. The results demonstrated that the extraneous noise inherent in the data was effectively eliminated by NL-SGS-D_2_ hybrid preprocessing strategy, thereby enhancing the predictive performance of the model.

### 3.3. Optimizing the CNN Calibration Model

The spectral data were processed by the NL-SGS-D_2_ hybrid preprocessing strategy and a CNN model (NL-SGS-D_2_-CNN) was established. To obtain a higher performance calibration model, using R^2^_v_ and RMSEV of model as metrics, the parameters of the CNN were optimized, including the convolutional layer configurations, maximum epochs, and dropout implementation. Optimization results have been tabulated in Table 2. The Loss and RMSE plots during the optimization process were presented in Figures S4–S6.

During optimization, model performance initially improved with increasing epoch (60–100), with the highest R^2^_V_ of 0.9373 at 100 epochs. However, further optimization beyond this point led to a decline in R^2^_V_ to 0.9179, indicating that the model began memorizing noise artifacts. It was demonstrated in Figure S4A–H that an increase in the number of epochs led to a larger discrepancy between the training and validation curves, resulting in overfitting of the model. When dropout was being optimized to mitigate model overfitting, the epoch parameter was fixed at its optimized value. The model R^2^_V_ values increased from 0.9373 to 0.9384 after the addition of dropout regularization. The improvement stemmed from suppressing neuronal coadaptation and reducing overdependence on specific neurons by the application of dropout [38]. In summary, the CNN model was configured with a maximum of 100 training epochs with dropout.

**Figure 4:**
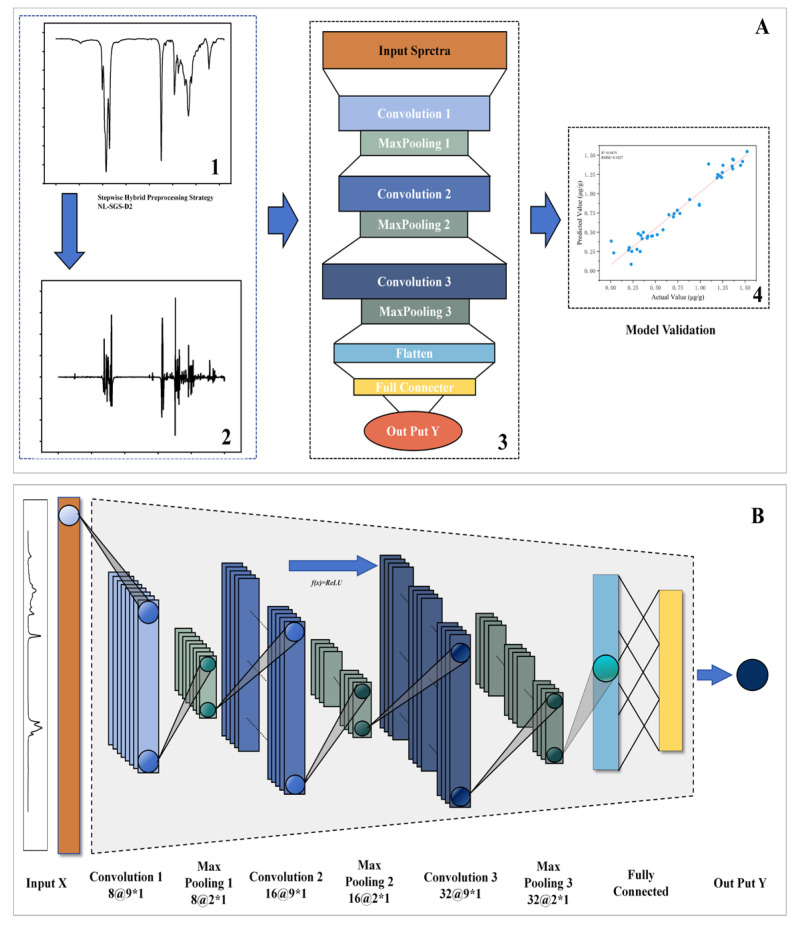
Schematic representation of CNN for quantitation of 3-MCPD in edible oil samples. (**A**) Schematic diagram of the NL-SGS-D_2_-CNN; (**B**) detailed illustration of the CNN architecture.

In addition to the two parameters mentioned above, the depth of convolutional layers was a critical factor in determining the model’s ability to learn data features. As the number of convolutional layers increased from 1 to 3, the R^2^_V_ data increase from 0.9384 to 0.9464. And, with further increases in the convolutional ranging from 4 to 5, the R^2^_V_ declined to 0.9304. The reason was because that the efficacy of spectral feature extraction from FTIR data depends on a harmonized CNN architecture depth [39]. Statistical analyses revealed that the most appropriate model parameters were set as follows: three convolutional layers was used, with dropouts and a maximum of 100 epochs. The CNN process for quantitative detection of 3-MCPD in edible oil samples is shown in Figure 4A. The CNN model architecture is depicted in Figure 4B. The convolutional kernel size was set to 9 × 1, with the number of filters per layer being 8, 16, and 32, respectively. The R^2^_V_ and RMSEV of the CNN model are 0.9464 and 0.0985, respectively.

### 3.4. Visualization of the NL-SGS-D_2_-CNN

**Figure 5:**
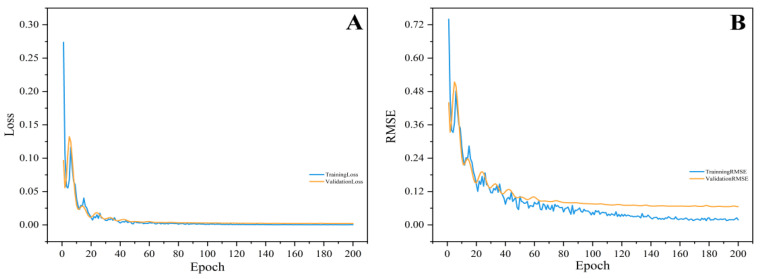
The variation curves of Loss (**A**) and RMSE (**B**) were demonstrated for the CNN model developed to predict 3-MCPD content.

The training dynamics during the CNN constructed were shown in Figure 5, including the progressive changes in Loss (Figure 5A) and RMSE (Figure 5B). It could be seen that as the model iterated, the RMSE value and the model’s Loss value were gradually decreased, and eventually returned to convergence, with the performance of model being gradually improved. The largest change in model performance was observed within the first 40 epochs during the model training process. This improvement was attributed to the Adam algorithm’s adaptive learning rate adjustment, which facilitated substantial gradient updates during the early training phase [40]. The negligible divergence between the two curves demonstrates the model’s generalization capability, reflecting the efficacy of the CNN training protocol. These mechanisms collectively enhanced model convergence efficiency and robustness. Overall, the model exhibited strong training performance and stability.

### 3.5. Quantitative Analysis of 3-MCPD by NL-SGS-D_2_-CNN

**Figure 6:**
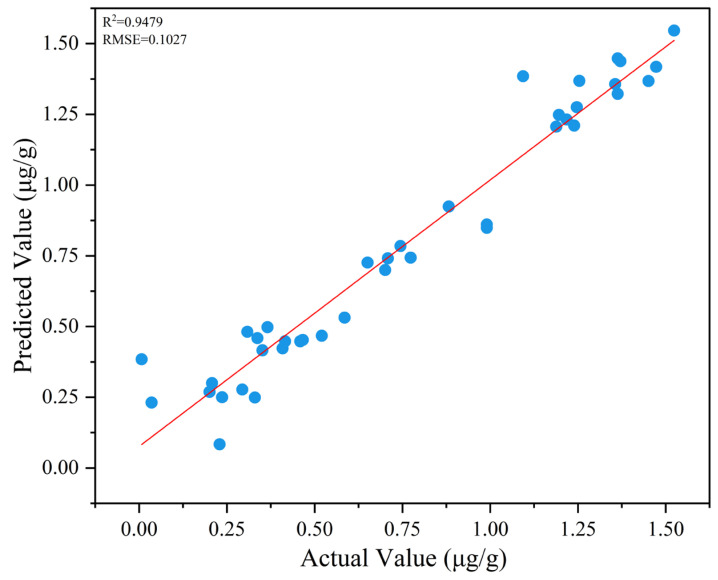
The relationship between the actual value and predicted value obtained by NL-SGS-D_2_-CNN model.

To rigorously evaluate the detection efficacy of the optimized CNN model in 3-MCPD quantitation, a dedicated prediction cohort was subjected to comprehensive validation. The detection performance of the hyperparameter-optimized CNN model for 3-MCPD concentration was presented in Figure 6. The R^2^ value for the prediction of 3-MCPD in actual fried samples was 0.9479, while the RMSE was 0.1027. The LOD and LOQ for 3-MCPD in this method were determined to be 0.3637 µg g^−1^ and 1.1021 µg g^−1^, respectively. The LOD was within the acceptable limit in the EU regulatory standard (1.25 μg g^−1^ for 3-MCPD in edible oils and fats) [41]. However, the LOQ of the NL-SGS-D_2_-CNN method was significantly higher than that of the AOCS method (0.002 μg g^−1^), but still complied with the detection criteria. Meanwhile, high model complexity was caused by the 3600-dimensional input. Therefore, further research was required in the future to improve the sensitivity of the method.

Nonetheless, the computed residual prediction deviation (RPD) value of 4.19 (RPD > 3) for the dataset demonstrates that the NL-SGS-D_2_-CNN model achieves reliable prediction of fried oil samples with varying 3-MCPD concentrations [42]. In general, after implementing the stepwise hybrid preprocessing strategy, the predictive error of the model was significantly reduced, which proved that the predictive performance of the model was more stable.

### 3.6. Comparison of Different Modeling Methods

**Figure 7:**
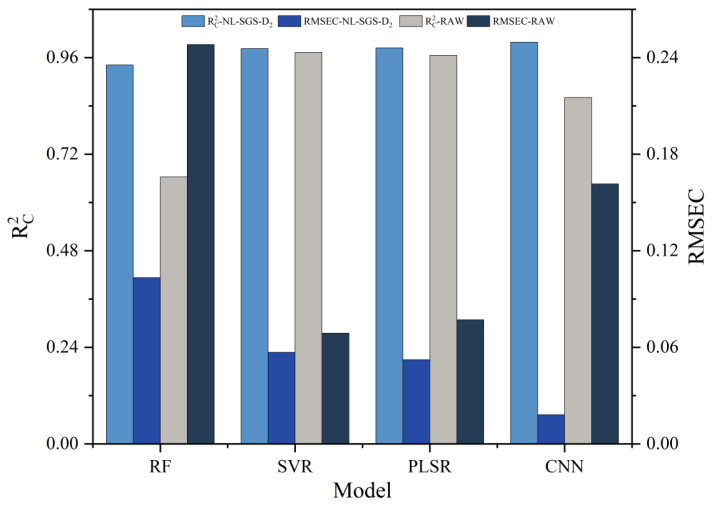
Comparison of performance among RF, SVR, PLSR, and CNN models.

To compare the feature extraction capabilities across models, and CNN architectures with hybrid preprocessed datasets was compared with other machine learning method including RF, SVR, and PLSR. Comparative performance metrics are detailed in Figure 7, while the model parameter specifications are provided in Table 3. The R^2^_C_ and R^2^_V_ of SVR model were 0.9823 and 0.8096. The performance of PLSR was comparable to SVR. The RF model exhibited the weakest overall performance, achieving an R^2^_C_ of 0.9416 and an R^2^_V_ of 0.9295. Compared to the above methods, the CNN model demonstrated the most balanced performance, achieving the highest R^2^_C_ value (0.9982) and R^2^_V_ value (0.9464). Overall, the CNN model was superior to traditional methods, at data fitting capabilities.

PLSR enhanced robustness by extracting principal components highly correlated with the dependent variable [43]. However, SVR’s handling of nonlinear data through kernel functions over-relied on the local structure of the data, which led to overfitting. For the RF model, decision tree splitting processes caused misjudgments of local optimality and the random subspace sampling approach resulted in insufficient sampling of spectral features and introduced bias [44]. In contrast, the CNN model adopted multi-layer feature extraction strategies to achieve progressive representation between signals and labels. The CNN model effectively distinguishing noise from features and suppressing model overfitting. Ultimately, the model performance was enhanced.

Besides the selection of model, the effectiveness of preprocessing strategies proved to be a key determinant in model performance. The stepwise hybrid preprocessing successfully enhanced the performance of all models, with 42% improvement for RF model, 1% for the SVR model, 2% for the PLSR model, and 16% for the CNN model. The most significant impact of preprocessing on RF model performance was attributed to its suppression of noise-induced variance in decision tree ensembles. For the CNN model, the performance enhancement (R^2^c improved from 0.8607 to 0.9982) was driven by the NL-SGS-D_2_-CNN for effective extraction of spectral features for 3-MCP. In conclusion, NL-SGS-D_2_-CNN models exhibited significant potential in regression modeling approaches.

## 4. Conclusions

This study integrates NL-SGSL-D_2_ with CNN to quantify 3-MCPD in fried oils based on FTIR spectroscopy. The combination of NL-SGS-D_2_ with CNN can realize the noise elimination and multiscale discriminative fingerprint feature extraction in one model and remedy the limitations of conventional analytical method. Under the optimal parameter setting, NL-SGS-D_2_-CNN achieved an R^2^_C_ of 0.9982 and RMSEC of 0.0181 on the training set. This represents a 16% performance improvement compared to the without NL-SGS-D_2_. The LOD (0.3637 µg g^−1^) and LOQ (1.1021 µg g^−1^) of the proposed method were within the permissible range for the EU 3-MCPD regulatory standard (1.25 µg g^−1^). In conclusion, a rapid and prospectively applicable analytical approach was established in this study by integrating spectroscopic techniques with deep learning methodologies for quantifying 3-MCPD in edible oils. The hybrid preprocessing strategy proposed in this study can be integrated with portable detection devices to establish the foundation for rapid real-time monitoring of oil quality control. However, the NL-SGS-D_2_-CNN method still has issues such as high data dimensionality and poor sensitivity. These two aspects will be prioritized in future research to enhance the method’s performance.

## Figures and Tables

**Table 1:** The pretreatment methods used in the stepwise hybrid preprocessing strategy.

NO.	Method	NO.	Method	NO.	Method
1	RAW	13	SGS	25	NL-SNV
2	RAW-D_1_	14	SGS-D_1_	26	NL-SNV-D_1_
3	RAW-D_2_	15	SGS-D_2_	27	NL-SNV-D_2_
4	MSC	16	SGS-MSC	28	NL-SGS
5	MSC-D_1_	17	SGS-MSC-D_1_	29	NL-SGS-D_1_
6	MSC-D_2_	18	SGS-MSC-D_2_	30	NL-SGS-D_2_
7	SNV	19	SGS-SNV	31	NL-SGS-MSC
8	SNV-D_1_	20	SGS-SNV-D_1_	32	NL-SGS-MSC-D_1_
9	SNV-D_2_	21	SGS-SNV-D_2_	33	NL-SGS-MSC-D_2_
10	NL	22	NL-MSC	34	NL-SGS-SNV
11	NL-D_1_	23	NL-MSC-D_1_	35	NL-SGS-SNV-D_1_
12	NL-D_2_	24	NL-MSC-D_2_	36	NL-SGS-SNV-D_2_

**Table 2:** The performance results of the CNN model was recorded during different phases of hyperparameter optimization.

Convolutional Layer	Dropout	Max Number of Epochs	R^2^_C_	RMSEC	R^2^_V_	RMSEV
1	No	60	0.9675	0.0781	0.9257	0.1160
1	No	80	0.9790	0.0628	0.9335	0.1097
1	No	100	0.9868	0.0497	0.9373	0.1065
1	No	120	0.9967	0.0241	0.9179	0.1149
1	YES	100	0.9859	0.0515	0.9384	0.1056
2	YES	100	0.9959	0.0279	0.9302	0.1124
3	YES	100	0.9982	0.0181	0.9464	0.0985
4	YES	100	0.9993	0.0117	0.9436	0.1010
5	YES	100	0.9823	0.0575	0.9304	0.1122

**Table 3:** Comparative performance evaluation of RF, SVR, PLSR, and CNN models.

Model	Methods	Calibration Set		Validation Set	
		R^2^_C_	RMSEC	R^2^_V_	RMSEV
RF	NL-SGS-D_2_	0.9416 ^cd^	0.1033 ^bc^	0.9295 ^a^	0.1176 ^cd^
	RAW	0.6636 ^e^	0.2481 ^a^	0.5424 ^d^	0.2995 ^a^
SVR	NL-SGS-D_2_	0.9823 ^ab^	0.0569 ^de^	0.8096 ^b^	0.1932 ^bc^
	RAW	0.9727 ^bc^	0.0688 ^cd^	0.6705 ^c^	0.2440 ^ab^
PLSR	NL-SGS-D2	0.9842 ^ab^	0.0523 ^e^	0.9485 ^a^	0.1101 ^de^
	RAW	0.9656 ^bc^	0.0771 ^cd^	0.9435 ^a^	0.0997 ^ef^
CNN	NL-SGS-D_2_	0.9982 ^a^	0.0181 ^f^	0.9464 ^a^	0.0985 ^f^
	RAW	0.8607 ^d^	0.1615 ^ab^	0.5607 ^d^	0.2819 ^a^

Note: Different letters denote significant differences in the performance of models established by different preprocessing methods (*p* < 0.05).

## Data Availability

The original contributions presented in this study are included in the article/Supplementary Material. Further inquiries can be directed to the corresponding author.

## Supplementary Materials

The following supporting information can be downloaded at: https://www.mdpi.com/article/10.3390/foods14101670/s1, Figure S1: Hybrid preprocessing strategy without derivative for FTIR spectra at 400 cm^−1^ to 4000 cm^−1^. (1. RAW; 2. MSC; 3. SNV; 4 NL; 5. SGS; 6. SGS-MSC; 7. SGS-SNV; 8. NL-MSC; 9. NL-SNV; 10. NL-SGS; 11. NL-SGS-MSC; 12 NL-SGS-SNV); Figure S2: Hybrid preprocessing strategy with D_1_ for FTIR spectra at 400 cm^−1^ to 4000 cm^−1^. (1. RAW-D_1_; 2. MSC-D_1_; 3. SNV-D_1_; 4. NL-D_1_; 5. SGS-D_1_; 6. SGS-MSC-D_1_; 7. SGS-SNV-D_1_; 8. NL-MSC-D_1_; 9. NL-SNV-D_1_; 10. NL-SGS-D_1_; 11. NL-SGS-MSC-D_1_; 12 NL-SGS-SNV-D_1_); Figure S3: Hybrid preprocessing strategy with D_2_ for FTIR spectra at 400 cm^−1^ to 4000 cm^−1^. (1. RAW-D_2_; 2. MSC-D_2_; 3. SNV-D_2_; 4. NL-D_2_; 5. SGS-D_2_; 6. SGS-MSC-D_2_; 7. SGS-SNV-D_2_; 8. NL-MSC-D_2_; 9. NL-SNV-D_2_; 10. NL-SGS-D_2_; 11. NL-SGS-MSC-D; 12 NL-SGS-SNV-D_2_); Figure S4: The variation plots of Loss and RMSE during the maximum Epoch number optimization process were as follows. (A. Loss plot when the maximum Epoch number was 60; B. RMSE plot when the maximum Epoch number was 60; C. Loss plot when the maximum Epoch number was 80; D. RMSE plot when the maximum Epoch number was 80; E. Loss plot when the maximum Epoch number was 100; F. RMSE plot when the maximum Epoch number was 100; G. Loss plot when the maximum Epoch number was 120; H. RMSE plot when the maximum Epoch number was 120); Figure S5: The variation plots of Loss and RMSE during the epoch optimization process were as follows.(A. Loss plot when used dropout; B. RMSE plot when used dropout; C. Loss plot without dropout; D. RMSE plot without dropout.); Figure S6: The variation plots of Loss and RMSE during the number of convolutional layers optimization process were as follows. (A. Loss plot with one convolutional layer; B. RMSE plot with one convolutional layer; C. Loss plot with two convolutional layers; D. RMSE plot with two convolutional layers; E. Loss plot with three convolutional layers; F. RMSE plot with three convolutional layers; G. Loss plot with four convolutional layers; H. RMSE plot with four convolutional layers; I. Loss plot with five convolutional layers; J. RMSE plot with five convolutional layers.); Table S1: Model performance under different hybrid preprocessing strategy methods.
