# Supplementary material for: Quantitative Analysis of 3-Monochloropropane-1,2-diol in Fried Oil Using Convolutional Neural Networks Optimizing with a Stepwise Hybrid Preprocessing Strategy Based on Fourier Transform Infrared Spectroscopy
Source: Foods. 2025 May 9;14(10):1670. doi: 10.3390/foods14101670 (PMC12111188; doi:10.3390/foods14101670)
Supplement: Supplementary file 1 [file foods-14-01670-s001.zip › foods-3579040-supplementary.pdf]

Supplementary material

Quantitative Analysis of 3-Monochloropropane-1,2-diol in Fried Oil Using Convolutional Neural Networks Optimizing with a Stepwise Hybrid Preprocessing Strategy Based on Fourier Transform Infrared Spectroscopy

Xi Wang [1], Siyi Wang [1], Shibing Zhang [1], Jiping Yin [2] and Qi Zhao [1,*]

[1] State Key Laboratory of Marine Food Processing and Safety Control, School of Food Science and Technology, Dalian Polytechnic University, Dalian 116034, China; wangxi9215@icloud.com (X.W.); www011478@163.com (S.W.); 13470306550@163.com (S.Z.)

[2] Information Technology Center, Dalian Polytechnic University, Dalian 116034, China; chinayinjp@dlpu.edu.cn

* Correspondence: zhaoqi@dlpu.edu.cn; Tel.: +86-411-86323453

Figure S1. Hybrid preprocessing strategy without derivative for FTIR spectra at 400 cm^{-1} to 4000 cm^{-1}. (1. RAW; 2. MSC; 3. SNV; 4 NL.; 5. SGS; 6. SGS-MSC; 7. SGS-SNV; 8. NL-MSC; 9. NL-SNV; 10. NL-SGS; 11. NL-SGS-MSC; 12 NL-SGS-SNV)

Figure S2. Hybrid preprocessing strategy with D_1 for FTIR spectra at 400 cm^{-1} to 4000 cm^{-1}. (1. RAW-D_1; 2. MSC-D_1; 3. SNV-D_1; 4. NL-D_1; 5. SGS-D_1; 6. SGS-MSC-D_1; 7. SGS-SNV-D_1; 8. NL-MSC-D_1; 9. NL-SNV-D_1; 10. NL-SGS-D_1; 11. NL-SGS-MSC-D_1; 12 NL-SGS-SNV-D_1)

Figure S3. Hybrid preprocessing strategy with D_2 for FTIR spectra at 400 cm^{-1} to 4000 cm^{-1}. (1. RAW-D_2; 2. MSC-D_2; 3. SNV-D_2; 4. NL-D_2; 5. SGS-D_2; 6. SGS-MSC-D_2; 7. SGS-SNV-D_2; 8. NL-MSC-D_2; 9. NL-SNV-D_2; 10. NL-SGS-D_2; 11. NL-SGS-MSC-D; 12 NL-SGS-SNV-D_2)

Figure S4. The variation plots of Loss and RMSE during the maximum Epoch number optimization process were as follows.(A. Loss plot when the maximum Epoch number was 60; B. RMSE plot when the maximum Epoch number was 60; C. Loss plot when the maximum Epoch number was 80; D. RMSE plot when the maximum Epoch number was 80; E. Loss plot when the maximum Epoch number was 100; F. RMSE plot when the maximum Epoch number was 100; G. Loss plot when the maximum Epoch number was 120; H. RMSE plot when the maximum Epoch number was 120).

Figure S5. The variation plots of Loss and RMSE during the epoch optimization process were as follows. (A. Loss plot when used dropout; B. RMSE plot when used dropout; C. Loss plot without dropout; D. RMSE plot without dropout.)

Figure S6. The variation plots of Loss and RMSE during the number of convolutional layers optimization process were as follows. (A. Loss plot with one convolutional layer; B. RMSE plot with one convolutional layer; C. Loss plot with two convolutional layers;

D. RMSE plot with two convolutional layers; E. Loss plot with three convolutional layers; F. RMSE plot with three convolutional layers; G. Loss plot with four convolutional layers; H. RMSE plot with four convolutional layers; I. Loss plot with five convolutional layers; J. RMSE plot with five convolutional layers.)

Table S1. Model performance under different hybrid preprocessing strategy methods.

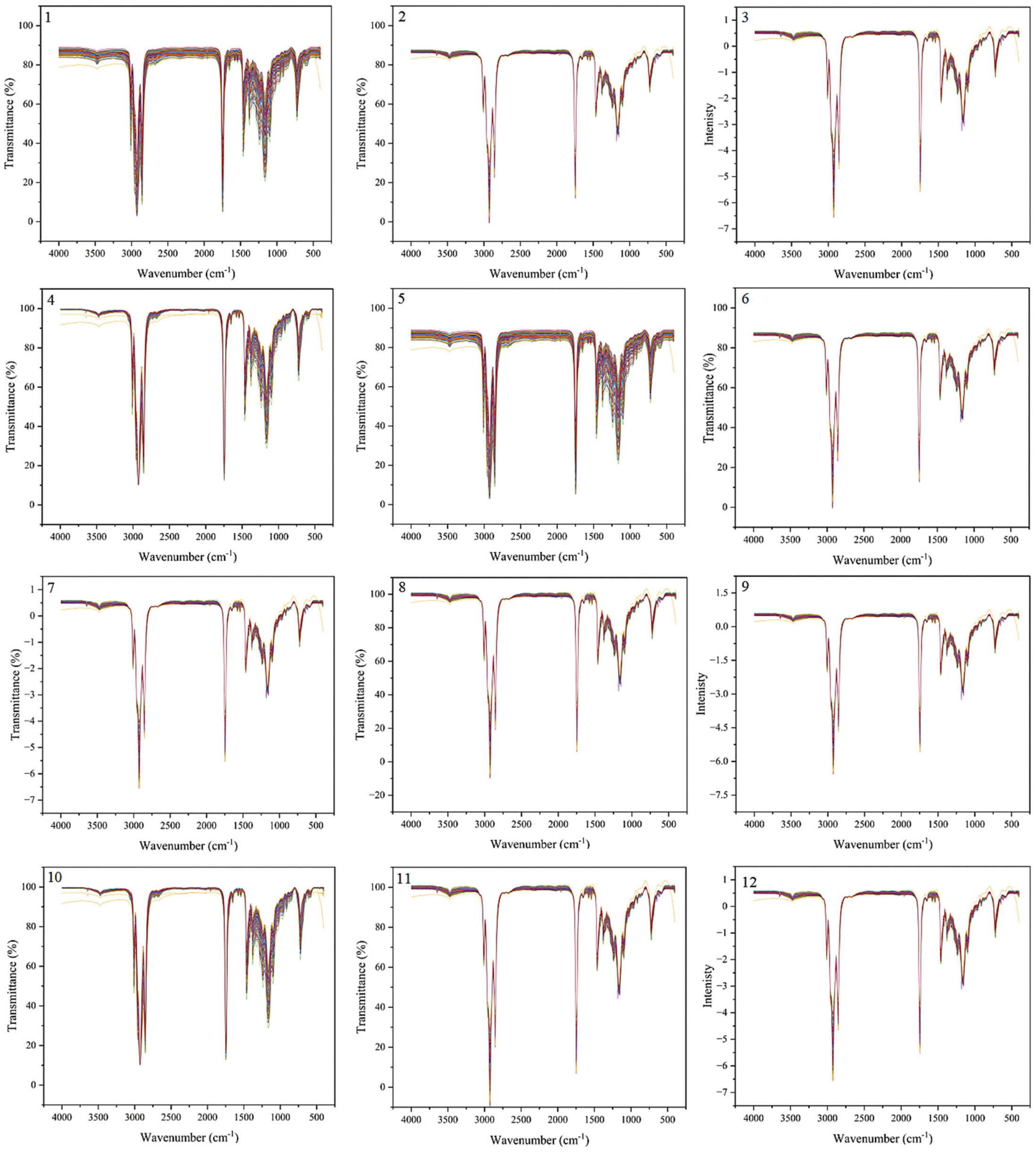

Figure S1. FTIR spectra subjected to preprocessed transformations. (1. RAW; 2. MSC; 3. SNV; 4 NL.; 5. SGS; 6. SGS-MSC; 7. SGS-SNV; 8. NL-MSC; 9. NL-SNV; 10. NL-SGS; 11. NL-SGS-MSC; 12 NL-SGS-SNV)

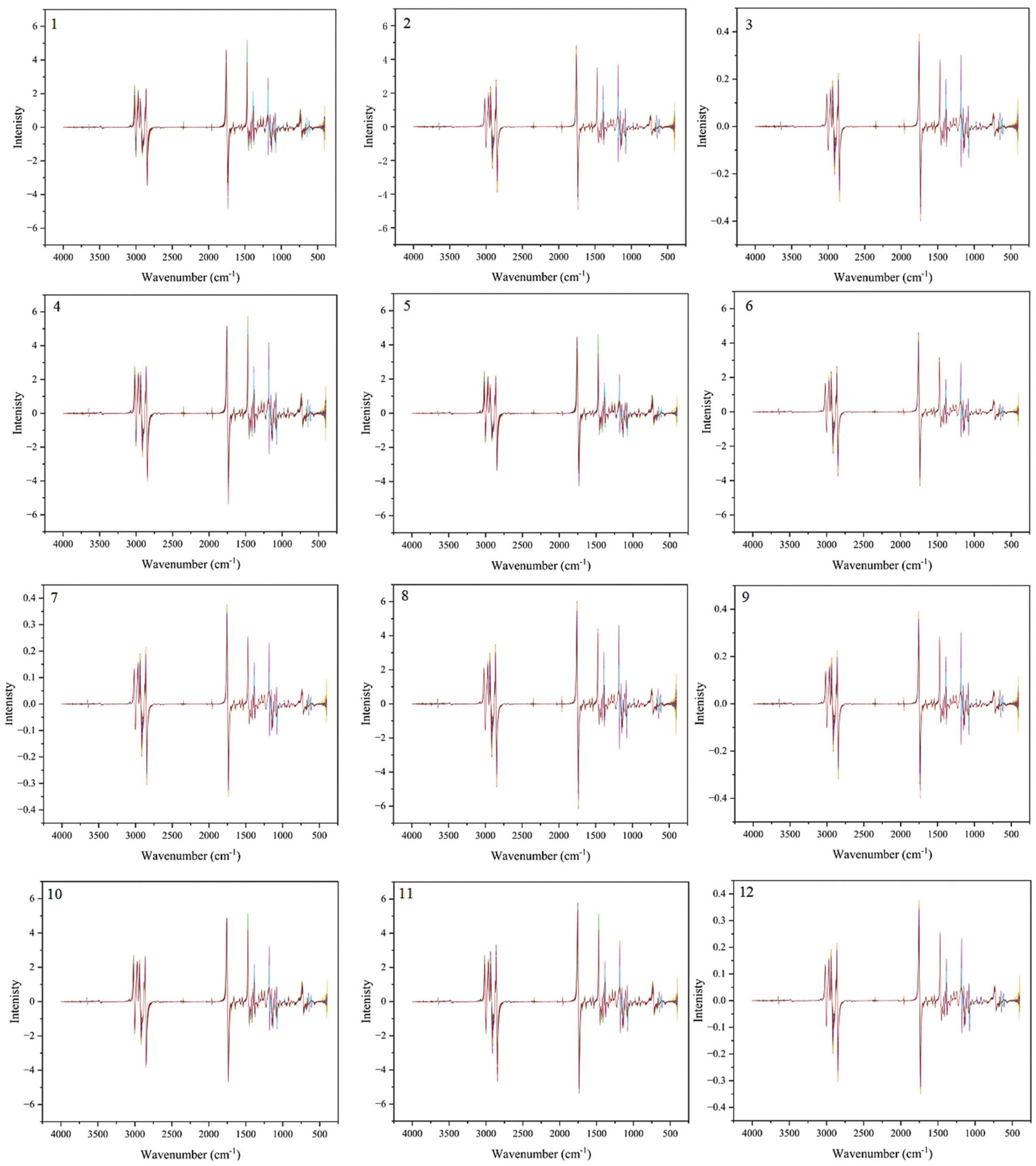

Figure S2. First-derivative(D_1) enhancement of preprocessed FTIR spectra. (1. RAW-D_1; 2. MSC-D_1; 3. SNV-D_1; 4. NL-D_1; 5. SGS-D_1; 6. SGS-MSC-D_1; 7. SGS-SNV-D_1; 8. NL-MSC-D_1; 9. NL-SNV-D_1; 10. NL-SGS-D_1; 11. NL-SGS-MSC-D_1; 12 NL-SGS-SNV-D_1)

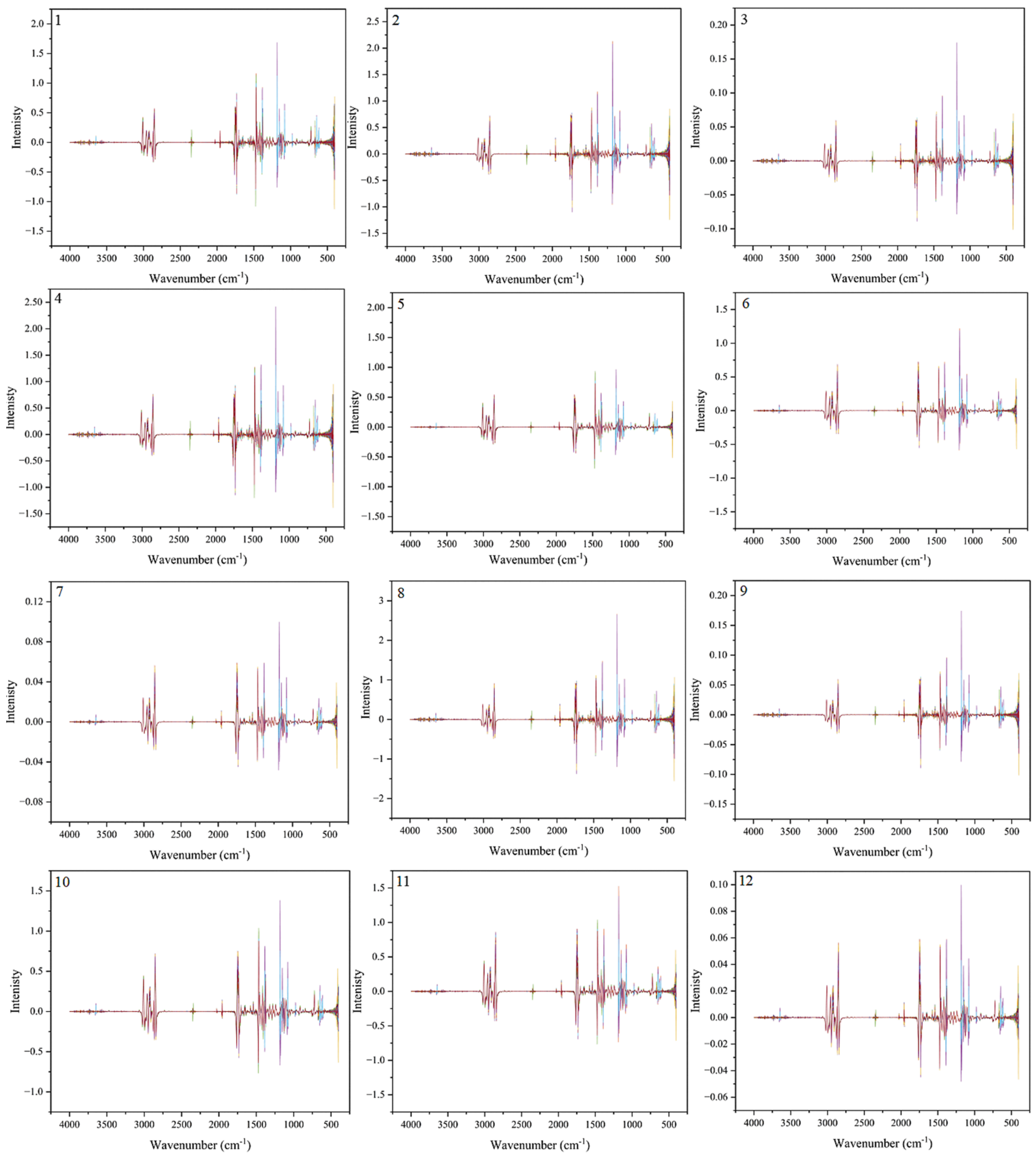

Figure S3. Second-derivative(D2) enhancement of preprocessed FTIR spectra. (1. RAW-D2; 2. MSC-D2; 3. SNV-D2; 4. NL-D2; 5. SGS-D2; 6. SGS-MSC-D2; 7. SGS-SNV-D2; 8. NL-MSC-D2; 9. NL-SNV-D2; 10. NL-SGS-D2; 11. NL-SGS-MSC-D; 12 NL-SGS-SNV-D2)

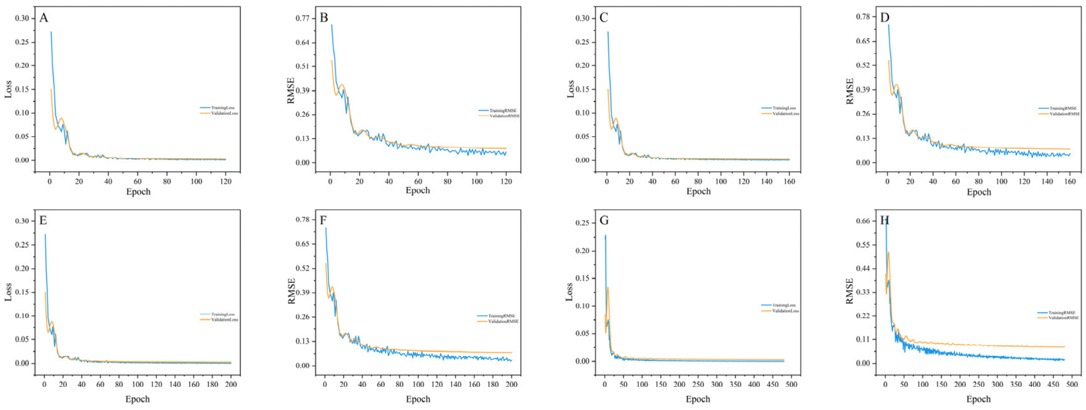

Figure S4. The variation plots of Loss and RMSE during the maximum Epoch number optimization process were as follows.(A. Loss plot when the maximum Epoch number was 60; B. RMSE plot when the maximum Epoch number was 60; C. Loss plot when the maximum Epoch number was 80; D. RMSE plot when the maximum Epoch number was 80; E. Loss plot when the maximum Epoch number was 100; F. RMSE plot when the maximum Epoch number was 100; G. Loss plot when the maximum Epoch number was 120; H. RMSE plot when the maximum Epoch number was 120).

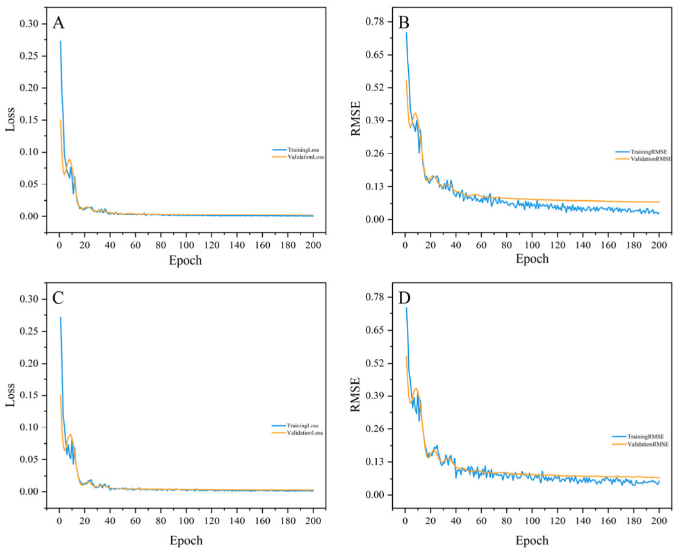

Figure S5. The variation plots of Loss and RMSE during the epoch optimization process were as follows. (A. Loss plot when used dropout; B. RMSE plot when used dropout; C. Loss plot without dropout; D. RMSE plot without dropout.)

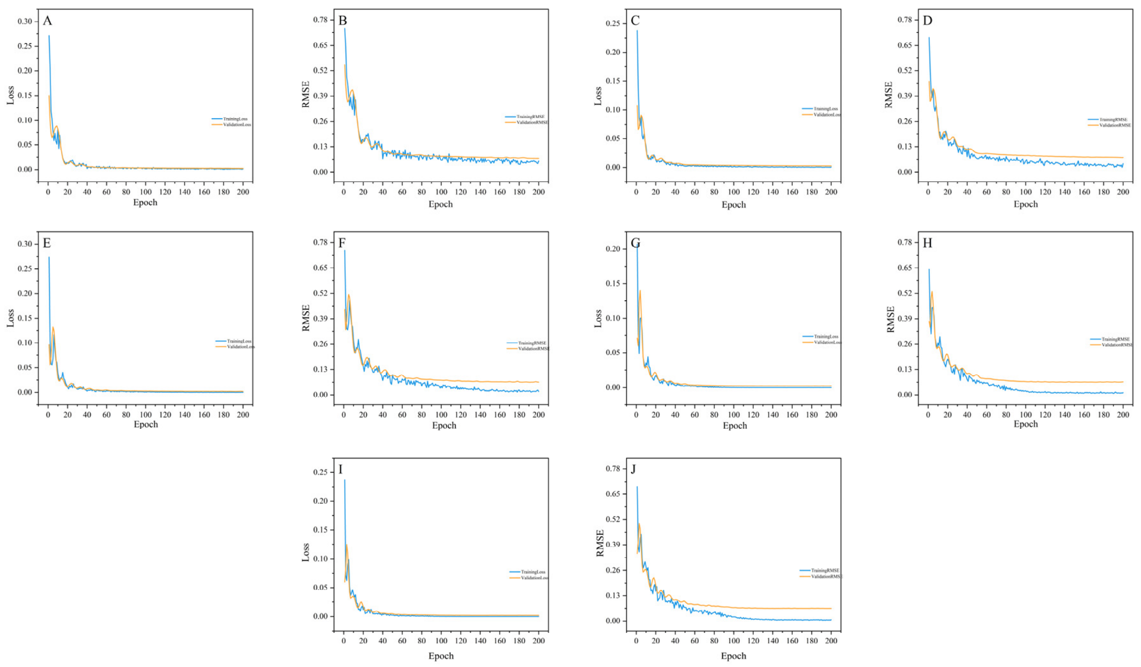

Figure S6. The variation plots of Loss and RMSE during the number of convolutional layers optimization process were as follows. (A. Loss plot with one convolutional layer; B. RMSE plot with one convolutional layer ; C. Loss plot with two convolutional layers; D. RMSE plot with two convolutional layers; E.Loss plot with three convolutional layers; F. RMSE plot with three convolutional layers; G.Loss plot with four convolutional layers; H. RMSE plot with four convolutional layers; I. Loss plot with five convolutional layers; J. RMSE plot with five convolutional layers.)

Table S1. Model performance under different preprocessing methods.

Number	Calibration Set		Validation Set	
	R^2C	RMSEC	R^2V	RMSEV
1	0.9656	0.0771	0.9435	0.0997
2	0.9685	0.0738	0.9402	0.1023
3	0.9746	0.0663	0.9464	0.0973
4	0.9456	0.0970	0.9282	0.1124
5	0.9657	0.0771	0.9346	0.1072
6	0.9742	0.0668	0.9412	0.1017
7	0.9501	0.0929	0.9365	0.1059
8	0.9663	0.0764	0.9372	0.1045
9	0.9745	0.0664	0.9419	0.1009
10	0.9657	0.0771	0.9384	0.1043
11	0.9686	0.0738	0.9432	0.0996
12	0.9773	0.0619	0.9452	0.0979
13	0.9782	0.0614	0.9637	0.0799
14	0.9742	0.0669	0.9479	0.0958
15	0.9713	0.0705	0.9488	0.0950
16	0.9444	0.0981	0.9265	0.1135
17	0.9725	0.0690	0.9380	0.1046
18	0.9731	0.0682	0.9443	0.0987
19	0.9488	0.0941	0.9326	0.1088
20	0.9728	0.0686	0.9446	0.0989
21	0.9735	0.0677	0.9449	0.0983
22	0.9464	0.0963	0.9334	0.1086
23	0.9660	0.0767	0.9378	0.1046
24	0.9746	0.0663	0.9387	0.1039
25	0.9508	0.0923	0.9366	0.1052
26	0.9769	0.0632	0.9465	0.0967
27	0.9749	0.0659	0.9417	0.1008
28	0.9730	0.0683	0.9458	0.0982
29	0.9745	0.0664	0.9438	0.0995
30	0.9842	0.0523	0.9486	0.0951
31	0.9452	0.0974	0.9292	0.1116
32	0.9727	0.0687	0.9416	0.1016
33	0.9737	0.0675	0.9452	0.0978
34	0.9399	0.1020	0.9274	0.1125
35	0.9730	0.0684	0.9415	0.1013
36	0.9740	0.0670	0.9460	0.0973
